# Characterization of *Enterococcus faecalis* in different culture conditions

**DOI:** 10.1038/s41598-020-78998-5

**Published:** 2020-12-14

**Authors:** Mi-Ah Kim, Vinicius Rosa, Kyung-San Min

**Affiliations:** 1 Department of Conservative Dentistry, Jeonbuk National University, 567 Baekje-daero, Jeonju-si, 54896 Korea; 2 Discipline of Oral Sciences, Faculty of Dentistry, National University of Singapore, 9 Lower Kent Ridge Road, Singapore, 119085 Singapore; 3 Research Institute of Clinical Medicine, Jeonbuk National University, 20 Geonji-ro, Jeonju-si, 54907 Korea; 4 Biomedical Research Institute, Jeonbuk National University Hospital, 20 Geonji-ro, Jeonju, 54907 Korea

**Keywords:** Microbiology, Molecular biology

## Abstract

The aim of this study was to investigate how carbohydrates (glucose or sucrose) affect the characteristics of *Enterococcus faecalis (E. faecalis)* planktonic and biofilm in vitro. For this study, *E. faecalis* was cultured in tryptone-yeast extract broth with 0% glucose + 0% sucrose, 0.5% glucose, 1% glucose, 0.5% sucrose, or 1% sucrose. Viability of *E. faecalis* was examined by colony forming unit counting assays. Biofilm formation was assessed by measuring extracellular DNA (eDNA), a component of the biofilm matrix. Quantitative real-time PCR (qRT-PCR) was performed to investigate the expression of virulence-associated genes. Field emission scanning electron microscopy analysis, confocal laser scanning microscopy analysis, and crystal violet colorimetric assay were conducted to study *E. faecalis* biofilms. *E. faecalis* showed the highest viability and eDNA levels in 1% sucrose medium in biofilms. The result of qRT-PCR showed that the virulence-associated genes expressed highest in 1% sucrose-grown biofilms and in 1% glucose-grown planktonic cultures. *E. faecalis* showed highly aggregated biofilms and higher bacteria and exopolysaccharide (EPS) bio-volume in sucrose than in 0% glucose + 0% sucrose or glucose. The results indicate that the production of eDNA and EPS and expression of virulence-associated genes in *E. faecalis* are affected by the concentration of carbohydrates in biofilm or planktonic culture.

## Introduction

*Enterococcus faecalis* (*E. faecalis*) is commonly isolated from infected root canals and is regarded as one of the etiological agents associated with failed endodontic treatments^1–4^. *E. faecalis* can be frequently found in failed endodontic treatments with its ability to survive in an environment that lacks nutrients and in the tooth environment that is highly alkaline after treatment with intracanal medicaments^4–6^.

The pathogenicity and difficulty in removal of *E. faecalis* can be due to its ability to form biofilms since biofilms are related to 65% of bacterial infections and can be 1000-fold more resistant to antibiotics than planktonic cells^7–10^. In addition, *E. faecalis* biofilms formed on medical devices are known to cause some hospital-acquired infections^11,12^. Accordingly, the role of biofilm components including exopolysaccharides (EPS), proteins, and extracellular DNA (eDNA) in the biofilm matrix has been investigated^10,13–18^. Especially, the significance of eDNA in biofilms has been studied and demonstrated that it promoted and developed biofilm formation by aiding bacterial aggregation and biofilm stability^10,19,20^. Many researchers have also investigated virulence factors in clinically-isolated *E. faecalis* and reported that enterococcal surface protein (*esp*), gelatinase (*gelE*), aggregation substance (*asa*1), cytolysin B (*cylB*), and endocarditis-specific antigen A (*efaA*) gene, ArgR family transcription factor (*ahrC*), endocarditis and biofilm-associated pili (*ebpA*), enterococcal polysaccharide antigen (*epal*), epal and OG1RF_11715 (*epaOX*), and (p)ppGpp-synthetase/hydrolase (*relA*) genes were prevalent^21–30^.

One of the factors that affects biofilm formation is nutrient type and availability in the culture environment^31^. It has been reported that glucose significantly promotes biofilm formation of *E. faecalis* by inducing Esp synthesis^30^. However, the effect of glucose and sucrose supplementation on *E. faecalis* planktonic and biofilm phenotypes is still under-characterized. Therefore, we investigated the effects of different culture conditions on the characteristics of *E. faecalis* by examining the expression of genes associated with virulence, biofilm formation, and levels of eDNA, a biofilm matrix component.

## Results

### *Enterococcus faecalis* viability and eDNA production

**Figure 1:**
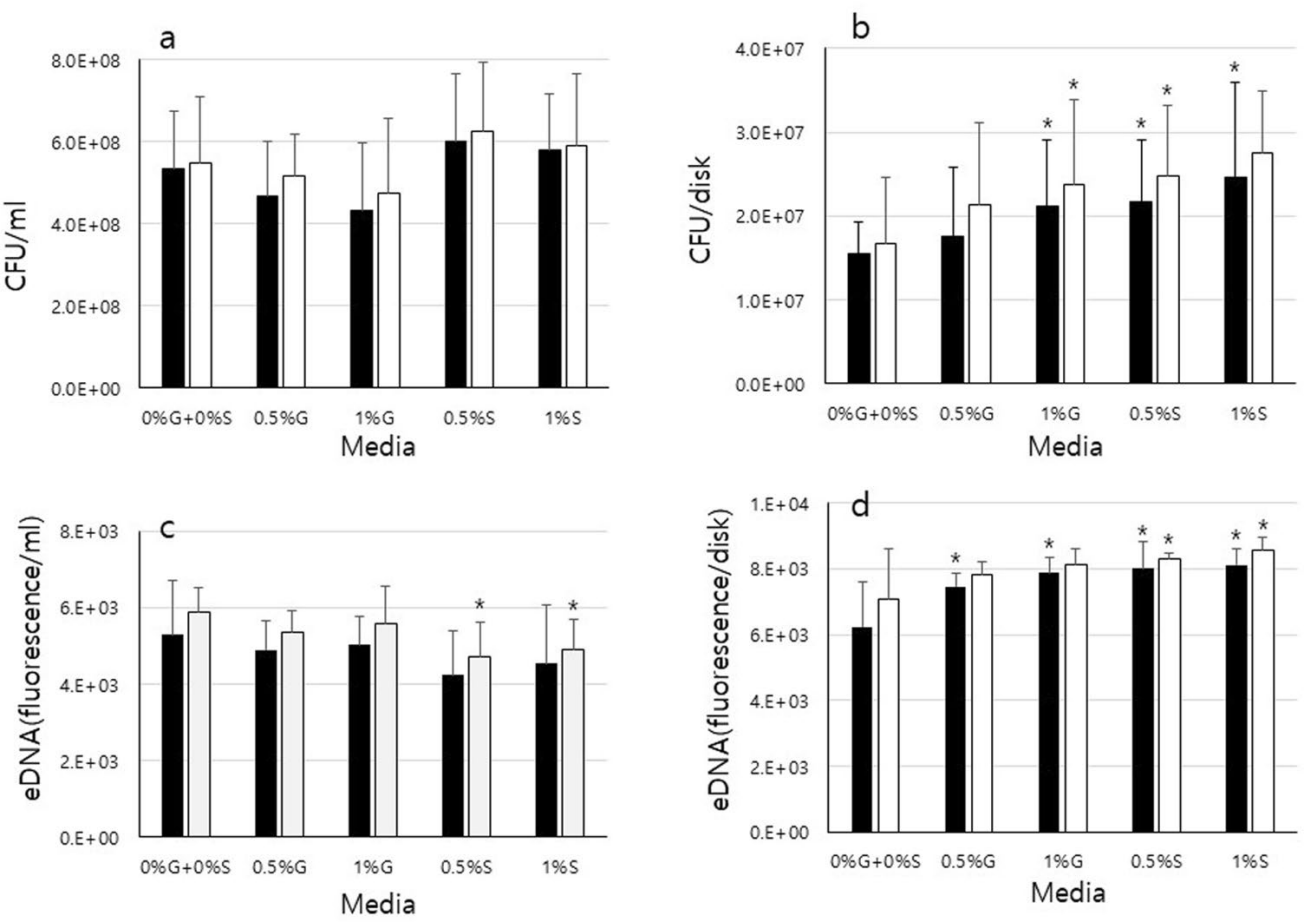
Analysis of *E. faecalis* grown in biofilms and planktonic culture. Viability and eDNA levels of ATCC29212 (black) and OG1RF (white) in (**a**,**c**) planktonic culture and (**b**,**d**) biofilms grown in 0% glucose + 0% sucrose (0%G + 0%S), 0.5% glucose (0.5%G), 1% glucose (1%G), 0.5% sucrose (0.5%S), or 1% sucrose (1%S) were examined. Data represent the mean and standard deviation of 3 independent experiments. Asterisks denote a statistically significant difference compared to 0%G + 0%S (*p* < 0.05).

The influence of media with or without carbohydrate (0% glucose + 0% sucrose, 0.5% glucose, 1% glucose, 0.5% sucrose, or 1% sucrose) on viability of *E. faecalis* in planktonic or biofilm conditions was investigated (Fig. 1a,b). *E. faecalis* showed higher colony-forming units (CFUs) in sucrose than in other media in both culture conditions, whereas it had lower CFUs in glucose in planktonic culture than in other media. The levels of eDNA were also examined since eDNA is one of the major components of the biofilm matrix (Fig. 1c,d). The highest level of eDNA was shown in 1% sucrose followed by 0.5% sucrose, 1% glucose 0.5% glucose, and 0% glucose + 0% sucrose in the biofilm condition (*p* < 0.05). In the planktonic condition, *E. faecalis* also had the highest level of eDNA in 0% glucose + 0% sucrose; however, it released more eDNA in glucose than in sucrose.

### Changes in gene expression of *E. faecalis* virulence-associated genes

**Figure 2:**
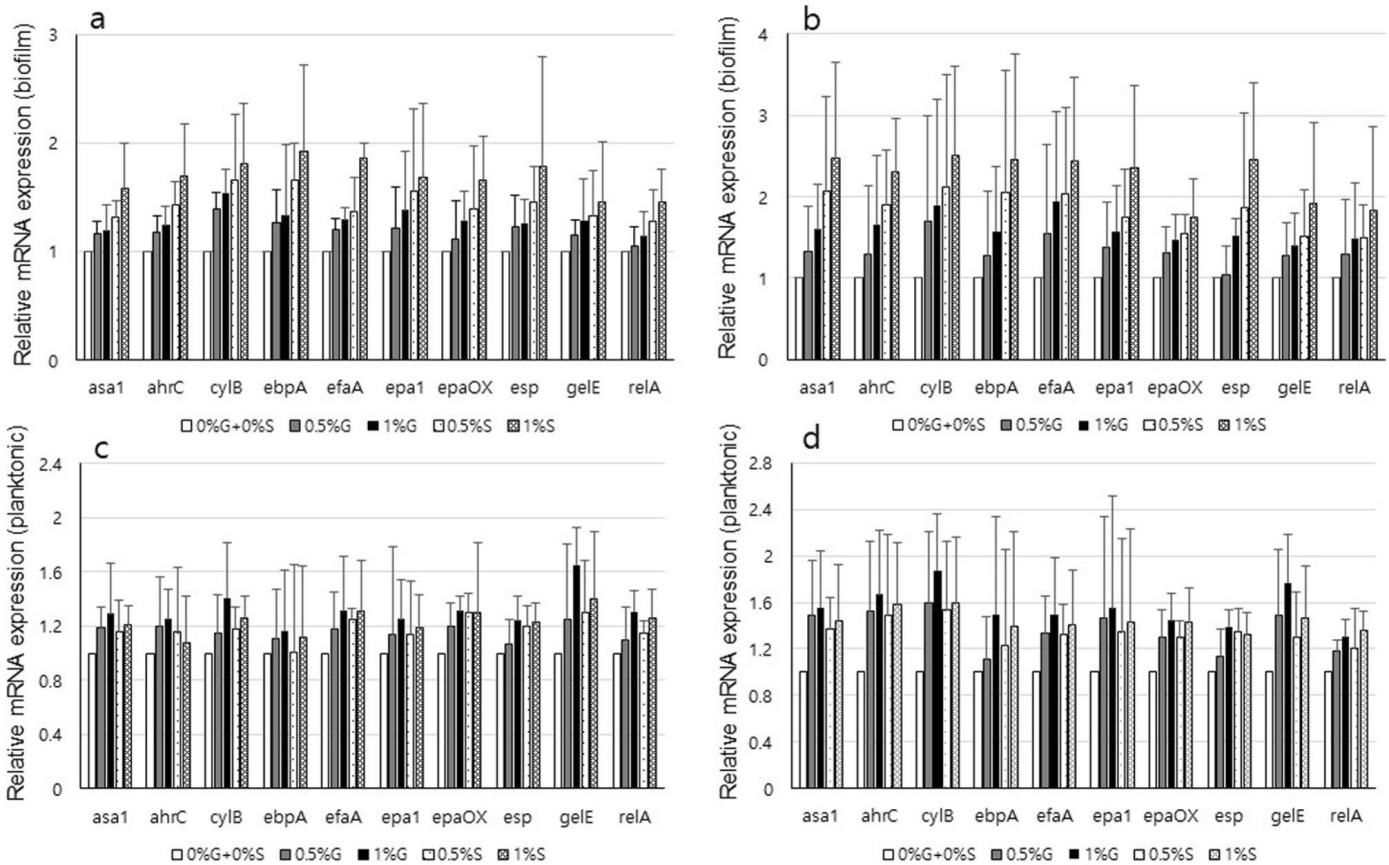
Expression of virulence-associated genes by qRT-PCR. RNA was prepared from *E. faecalis* (**a** and **c**) ATCC29212 and (**b** and **d**) OG1RF grown in 0% glucose + 0% sucrose (0%G + 0%S), 0.5% glucose (0.5%G), 1% glucose (1%G), 0.5% sucrose (0.5%S), or 1% sucrose (1%S). Data represent the mean and standard deviation of at least 3 independent experiments.

In biofilms, genes associated with *E. faecalis* virulence were expressed at significantly higher levels in sucrose, whereas their expression was upregulated in glucose in the planktonic growth condition (Fig. 2).

### EPS measurement and morphological observation of *E. faecalis* biofilms

**Figure 3:**
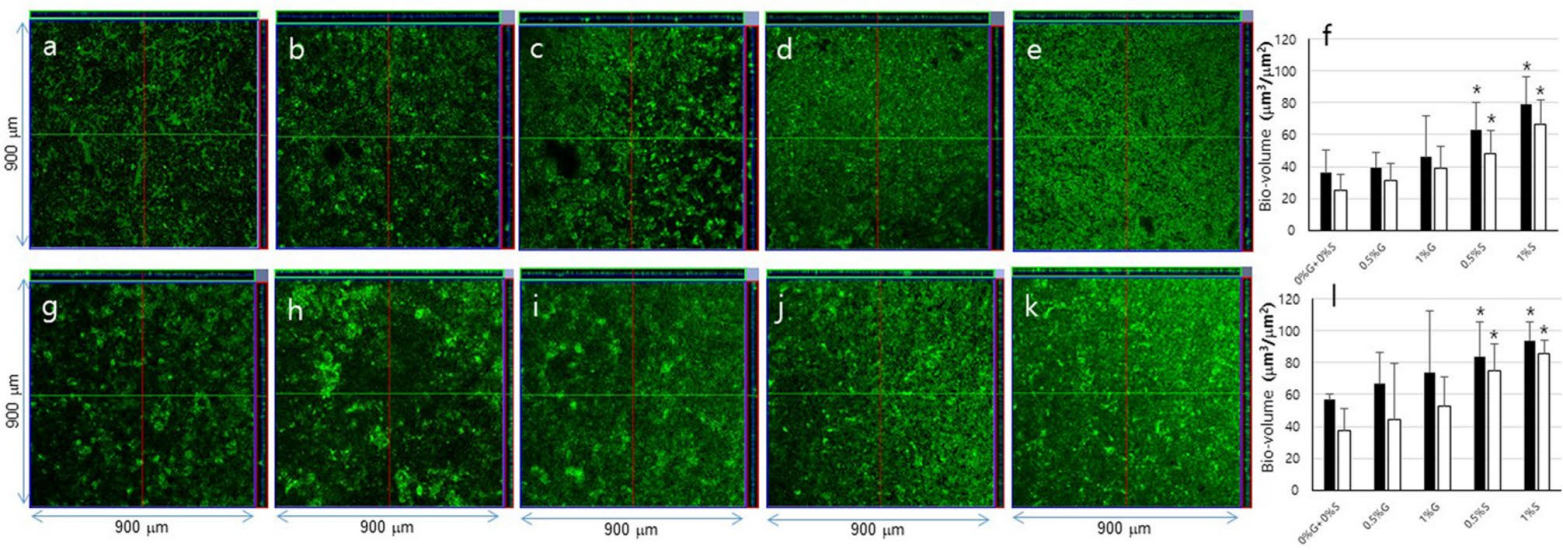
CLSM images of *E. faecalis* (**a**–**e**) ATCC29212 and (**g**–**k**) OG1RF biofilms. The bacteria were grown in 0% glucose + 0% sucrose (0%G + 0%S), 0.5% glucose (0.5%G), 1% glucose (1%G), 0.5% sucrose (0.5%S), or 1% sucrose (1%S). Bio-volume of bacteria (black) and EPS (white) of ATCC29212 (**f**) and OG1RF (**l**) biofilms were quantified by COMSTAT software (COMSTAT2, www.comstat.dk; Technical University of Denmark, Kongens Lyngby, Denmark). Data represent the mean and standard deviation of 3 independent experiments. Asterisks denote a statistically significant difference compared to 0%G + 0%S (*p* < 0.05).

**Figure 4:**
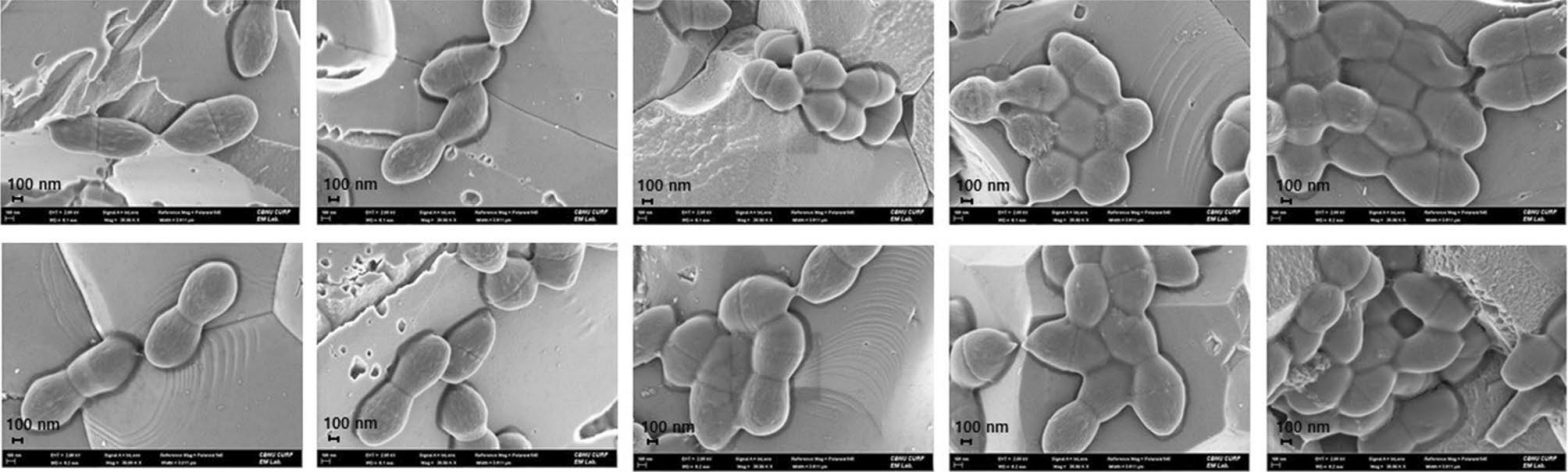
FE-SEM images of biofilms of *E. faecalis* (**a**–**e**) ATCC29212 and (**f**–**j**) OG1RF. *E. faecalis* biofilms were formed in 0% glucose + 0% sucrose (0%G + 0%S), 0.5% glucose (0.5%G), 1% glucose (1%G), 0.5% sucrose (0.5%S), or 1% sucrose (1%S) on the surface of hydroxyapatite (HA) disks. The images were examined with 30,000× magnification.

The effect of carbohydrate on *E. faecalis* biofilms was evaluated via bio-volume of bacteria and EPS using confocal laser scanning microscopy (CLSM) analysis (Fig. 3). *E. faecalis* showed the lowest level of bacteria and EPS bio-volumes in 0% glucose + 0% sucrose. The bacteria and EPS bio-volumes were higher in biofilms cultured in the presence of sucrose compared to biofilms cultured in glucose. As shown in the scanning electron microscopy (SEM) images in Fig. 4, *E. faecalis* biofilms aggregated to higher levels in sucrose compared to those in 0% glucose + 0% sucrose or glucose.

### Biofilm formation assay

**Figure 5:**
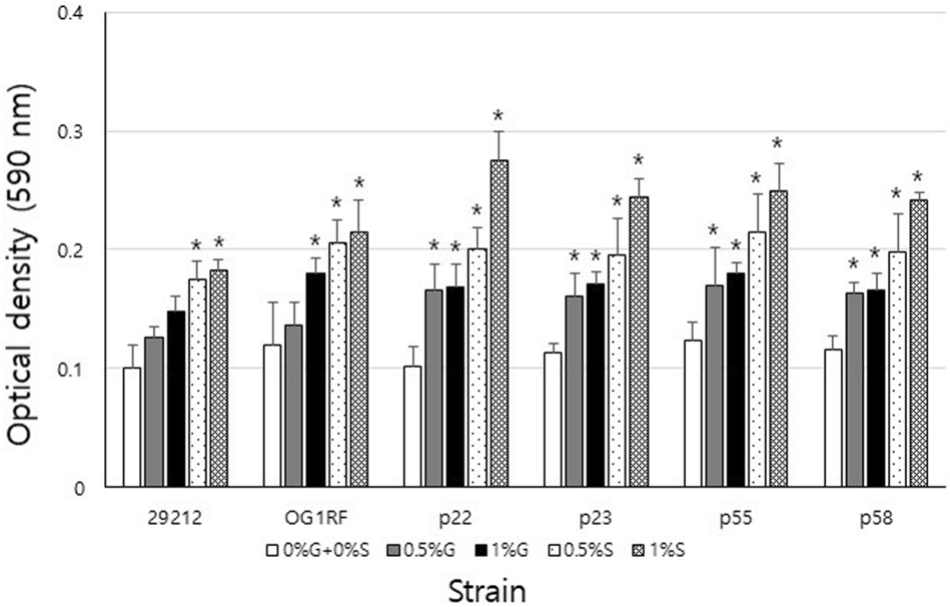
Biofilm formation of *E. faecalis* strains. Crystal violet staining was used to assay *E. faecalis* strain biofilm formation. The strains were cultured in 0% glucose + 0% sucrose (0%G + 0%S), 0.5% glucose (0.5%G), 1% glucose (1%G), 0.5% sucrose (0.5%S), or 1% sucrose (1%S). Data represent the mean and standard deviation of 3 independent experiments. Asterisks denote a statistically significant difference compared to 0%G + 0%S (*p* < 0.05).

Biofilm formation of *E. faecalis* ATCC29212, OG1RF and clinical strains was assessed by crystal violet colorimetric assay. As shown in Fig. 5, the strains showed higher biofilm formation in sucrose than in other media. OG1RF had greater biofilm mass than ATCC29212 in 0% glucose + 0% sucrose, 0.5% glucose, 1% glucose, 0.5% sucrose, or 1% sucrose, whereas clinical isolates formed larger biofilms than ATCC29212 and OG1RF in 1% sucrose.

## Discussion

*E. faecalis* is a pathogenic bacterium that can form biofilm, which leads to infectious disease, resistance to numerous antibiotics, and endodontic treatment failure^4,32,33^. Studies have shown that nutrients in culture media may affect bacterial biofilm formation^34,35^. Glucose was shown to enhance biofilm growth of *E. faecalis* by inducing Esp synthesis^30^. However, the results of the current study showed that the expressions of the *esp* gene along with other biofilm-associated genes such as asa1, cylB, efaA, ahrC, ebpA, epal, epaOX, and relA were higher in biofilms cultured in the presence of sucrose compared to glucose, whereas it showed higher or similar induction in glucose than in sucrose in planktonic culture. The results of this study suggested that *E. faecalis* could be more virulent in sucrose than in glucose with higher expression of the virulence-associated genes and greater biofilm formation in sucrose since many cases of bacterial infections are related to biofilms with 1000-fold more resistance against antibiotics than planktonic cells^7,8^. This needs further study.

Interestingly, although CFUs of *E. faecalis* were lower in sucrose than in 0% glucose + 0% sucrose in planktonic culture, it attached to the surface of HA disks more in 0% glucose + 0% sucrose than in glucose or sucrose in biofilm conditions when the disks were not washed (data not shown). However, when the disks were gently washed, unattached *E. faecalis* was easily removed from the disks and the result showed that the number of CFUs in 0% glucose + 0% sucrose was lower than that in the other media, whereas there was no significant difference of CFUs in biofilms formed in glucose or sucrose with or without washing. This could be the result of a lack of biofilm components such as eDNA in 0% glucose + 0% sucrose, as shown in Fig. 1d, that could give binding sites for *E. faecalis* and slower growth rates of bacteria in biofilms than in planktonic culture as demonstrated by Evans et al.^17,36^. In other words, *E. faecalis* could grow slowly in biofilms in sucrose since the bacteria aggregated more and formed compact biofilms in sucrose than in 0% glucose + 0% sucrose or glucose (Fig. 4).

Additional studies have investigated the role of eDNA, a component of the biofilm matrix, and demonstrated that it is essential for biofilm formation^20,37,38^. Thus, eDNA levels were examined and they were higher in biofilms in sucrose than in the other media. This result is in agreement with the result of a study that suggested that the eDNA levels in biofilms were relevant to the CFU numbers in the biofilms^39^. This could be explained by the higher expression of virulence-associated genes that were upregulated in those media and also could be the result of higher EPS bio-volume that could enmesh the bacteria with biofilm components such as eDNA. Interestingly, although the planktonic growth of *E. faecalis* in glucose was lower than that in 0% glucose + 0% sucrose or sucrose, their eDNA levels were similar to those collected from 0% glucose + 0% sucrose or sucrose in planktonic culture. This could be the result of higher expression of genes including some virulence-associated genes in glucose than in other media, as shown in Fig. 2. This needs further study.

The effect of sucrose on biofilm formation was extensively studied with *Streptococcus mutans* (*S. mutans*), one of the major agents that are associated with dental caries, and demonstrated that *S. mutans* utilized sucrose to produce glucans, an important component of biofilm matrix, leading to a high incidence of dental caries^40–42^. However, the effect of sucrose had not been studied with *E. faecalis*. According to the present study, *E. faecalis* produced higher levels of eDNA in sucrose and expressed more virulence-associated genes in biofilms than in other media. CLSM analysis showed that *E. faecalis* produced more EPS in sucrose, suggesting that *E. faecalis* could utilize sucrose and produce EPS. SEM analysis showed that *E. faecalis* aggregated more in the biofilm matrix in sucrose than in 0% glucose + 0% sucrose or glucose. *E. faecalis* biofilm formation of the strains was analyzed by crystal violet colorimetric assay and the result showed that *E. faecalis* formed larger biofilms in sucrose than in 0% glucose + 0% sucrose or glucose. The results of this study suggested that the biofilm formation ability of *E. faecalis* in sucrose could be significant clinically with greater biofilm formation and higher virulence-associated gene expression than in 0% glucose + 0% sucrose or glucose since infections of many bacteria are related to the ability of biofilm formation that induces more resistance against antibiotics than planktonic bacteria^7–9,34^.

In summary, *E. faecalis* produced more components of the biofilm matrix such as eDNA and EPS and had more highly expressed virulence-associated genes when grown in biofilms in sucrose relative to 0% glucose + 0% sucrose or glucose in biofilms. In planktonic culture, glucose-induced more eDNA/CFU and expression of virulence-associated genes than in 0% glucose + 0% sucrose or sucrose.

## Materials and methods

### Bacterial culture conditions

*E. faecalis* ATCC 29212 and ATCC 47077 (OG1RF) were grown overnight at 37 °C in brain heart infusion (BHI; Difco Laboratories, Detroit, MI, USA) media in 1% glucose as previously described with some modifications^39^. Briefly, 10 μl of the overnight cultured *E. faecalis* was cultured in 1 ml of BHI media for 2 h. For planktonic culture, *E. faecalis* (3 × 10^6^ CFU/mL) was cultured in a 24-well plate (SPL, Daejeon, Korea) in tryptone-yeast extract broth (TY) with 0% glucose + 0% sucrose, 0.5% glucose, 1% glucose, 0.5% sucrose, or 1% sucrose for 24 h without agitation at 37 °C. For biofilm formation, hydroxyapatite (HA) disks (2.93 cm^2^; Clarkson Chromatography Products, Inc. South Williamsport, PA, USA) were vertically placed in a 24-well plate that contained *E. faecalis* (3 × 10^6^ CFU/mL) in TY with 0% glucose + 0% sucrose, 0.5% glucose, 1% glucose, 0.5% sucrose, or 1% sucrose for 24 h without agitation at 37 °C. To prepare BHI plates, 1.5% (wt/vol) agar (Difco Laboratories) was added.

### Colony forming unit (CFU) assay and eDNA measurement

*E. faecalis* eDNA and CFUs were measured as previously described with some modifications^39^. Briefly, *E. faecalis* grown in planktonic culture was collected carefully and centrifuged at 10,000×*g* at 4 °C for 10 min. The pellet was resuspended in phosphate buffered saline (PBS) and sonicated using a sonifier (VCX 130 PB; Sonics and Materials Inc., Newtown, CT, USA) for 10 s at 7 W to separate eDNA from the bacteria. To collect *E. faecalis* in biofilms, HA disks were gently washed twice in PBS and sonicated in an ultrasonic bath (Power Sonic 410; Hwashin Technology Co., Seoul, Korea) for 10 min to break the biofilms in 2 ml PBS. The biofilms were then scraped using a spatula and sonicated with a sonifier for 10 s at 7 W. For CFU counting, an aliquot (0.1 ml) of the homogenized *E. faecalis* was serially diluted and plated on BHI agar plates and incubated at 37 °C. The remaining cells were centrifuged at 10,000×*g* at 4 °C for 10 min. The eDNA level was measured using a fluorescence microplate reader (HIDEX, Turku, Finland) at an excitation of 485 nm and emittance of 535 nm.

### Quantitative real-time PCR (qRT-PCR)

After 24 h incubation in planktonic culture and in biofilms, the bacteria were collected and sonicated. Then, the homogenized bacteria were treated using Trizol Max Bacterial RNA Isolation Kit (Life Technologies, Carlsbad, CA, USA) to extract RNA according to manufacturer’s instructions. Complementary DNA (cDNA) was amplified using a QuantiTech Reverse Transcription Kit (QIAGEN, Hilden, Germany). qRT-PCR was performed using a Rotor-Gene SYBR Green PCR Kit (QIAGEN, Hilden, Germany) and a Rotor-Gene Q real-time PCR machine (QIAGEN) with a 72-well rotor. The primers used in this study are shown in Table 1^43^. Relative expression was calculated by normalizing each test gene to the *E. faecalis* 16s RNA reference gene.

**Table 1:** Sequence of primers for qRT-PCR.

Name			Sequence		Size	Reference
16s rRNA	F	5′	CCGAGTGCTTGCACTCAATTGG	3′	138	Salah R, et al
	R	5′	CTCTTATGCCATGCGGCATAAAC	3′		
asa1	F	5′	ATG ACA AAC CCA AAG CCA AT	3′	260	This study
	R	5′	CGC AAT TCG TTC TAC ACC AA	3′		
gelE	F	5′	CGG ATT GGT TAC ACC ATT ATC C	3′	207	This study
	R	5′	TGC CAC TCC TTA TCC ATT TTT	3′		
cylB	F	5′	GCT CTA ATT GAC TCG GGG ATT	3′	180	This study
	R	5′	CAC TCT TGG AGC AAT CGT GT	3′		
esp	F	5′	TCG CTC CAA ATG AAA AAG ATG	3′	200	This study
	R	5′	CGG TTG AAC CTT CTT CTG GT	3′		
efaA	F	5′	ACC ATT AAG AAA TCA AAA GCA	3′	240	This study
	R	5′	CGT ATC GCC TTC TGT TCC TT	3′		
ahrC	F	5′	GTTGAACGTGTCGCCTTTTT	3′	233	This study
	R	5′	GCTTTTTCCTCGGATGATGA	3′		
ebpA	F	5′	AATTTCTGGAGAGAAGAATA	3′	175	This study
	R	5′	ACACACTCCCTTCTGGTCGT	3′		
relA	F	5′	AATGAAAGAACGCCTTCAGC	3′	215	This study
	R	5′	TGATTTTCTGGTGTTTCGTT	3′		
epal	F	5′	CAAATTATCCCGAGCCAGAA	3′	237	This study
	R	5′	AGAATTGCTGAGCCGACTTC	3′		
epaOX	F	5′	CAAGTAAACCTAGAGGGGAAA	3′	208	This study
	R	5′	TTCCCTAAAATTTTTCGATAC	3′		

### Field emission scanning electron microscopy (FE-SEM)

Biofilms formed on the HA disks in 0% glucose + 0% sucrose, 1% glucose, or 1% sucrose were fixed as previously described with some modifications^44^. In brief, HA disks were in 2.5% glutaraldehyde / 4% paraformaldehyde (Sigma-Aldrich, Saint Louis, MO, USA) in PBS at 4 °C overnight. They were then washed in PBS and a graded series of ethanol (25–100%) was used to dehydrate the samples. The samples were then air-dried, gold-coated, and observed using FE-SEM (Hitachi, Tokyo, Japan). The images were obtained on a Hitachi SU-70 using BSE detector with 10.0 kV voltage acceleration.

### Confocal laser scanning microscopy (CLSM)

CLSM analysis was performed to investigate the change of *E. faecalis* biofilms formed in media with different carbohydrates (0% glucose + 0% sucrose, 1% glucose, or 1% sucrose) as previously described^39^. *E. faecalis* (3 × 10^6^ CFU/ml) was cultured in a medium with 1 μM Alexa Fluor 647-labeled dextran conjugate (650/668 nm; Molecular Probes, Eugene, OR, USA), which was incorporated into the EPS matrix for 24 h at 37 °C. Then, *E. faecalis* on the HA disks were exposed to 2.5 μM SYTO 9 green fluorescent nucleic acid stain (480/500 nm; Molecular Probes) for 20 min at room temperature. Fifteen image stacks (512 × 512 pixel tagged image file format) were obtained using an LSM 510 META microscope (Carl Zeiss, Jena, Germany). The bio-volume of the bacteria and EPS was quantified using. The laser power was 5 mW for Alexa Fluor 647-labeled dextran conjugate (red channel—HeNe laser) and 20 mW for SYTO 9 (green channel—Ar laser). Three individual experiments were performed.

### Crystal violet assay

Crystal violet was used to assess the biofilm mass of *E. faecalis* ATCC29212 and OG1RF including clinical isolates as previously described with some modifications^10^. Briefly, *E. faecalis* (3 × 10^6^ CFU/ml) was cultured in a 96 well plate in TY with 0% glucose + 0% sucrose, 0.5% glucose, 1% glucose, 0.5% sucrose, or 1% sucrose for 24 h without agitation at 37 °C. Then the plate was washed with sterile water and added 0.5% crystal violet (Sigma-Aldrich, St. Louis, MO, USA) and kept at room temperature for 10 min. Then the plate was rinsed twice with sterile water, treated with 30% acetic acid (Fisher Scientific, Fair Lawn, NJ, USA), and measured at 590 nm (μQuant, Biotek Instrument, Winooski, VT, USA).

### Statistical analysis

All experiments were performed at least three times. The intergroup differences were estimated by one-way analysis of variance (ANOVA). The data are presented as mean and standard deviation (SD). A *p *value was considered as statistically significant when it was less than 0.05.

## Data Availability

The data generated and analyzed in this study are available from the corresponding author upon request.
